# The Association Between Discrimination and Self-Mastery in U.S. Chinese Older Adults

**DOI:** 10.1093/geroni/igaa057.1085

**Published:** 2020-12-16

**Authors:** Mailun Zhang, Mengting Li, XinQi Dong

**Affiliations:** 1 staff, New Brunswick, New Jersey, United States; 2 Rutgers University, New Brunswick, New Jersey, United States

## Abstract

Perceived discrimination related to one’s racial/ethnic membership has been linked to negative impact on the health and wellbeing of minority populations. While the anti-Chinese sentiment in the US dates to the 19th century, discrimination experienced by this population and its impact has been poorly understood. Self-mastery is a protective psychological resource reflecting one’s ability to cope with stressors. This study examines the interaction between discrimination and self-mastery among 3,157 US Chinese older adults. Data were obtained from the Population Study of Chinese Elderly in Chicago (PINE) collected between 2011 and 2013. Self-mastery was measured using the Pearlin Mastery Scale. Discrimination was measured using the Experiences of Discrimination instrument. Linear regression was used. Discrimination experiences were found common (21.3%) among the US Chinese older adults. Younger age, male gender, higher levels of education, higher income, being married, more children, and fewer medical comorbidities were associated with a higher sense of self-mastery. After controlling for these potential confounders, discrimination experiences appeared to be significantly associated with lower self-mastery. Specifically, people who have experienced discrimination when getting hired (Beta [B]=-4.47,Standard Error [SE]=1.04, p<0.01), in working environment (B=-1.13,SE=0.52, p<0.05), getting health care (B=-3.45,SE=0.85, p<0.01), getting services in a store or restaurant (B=-2.12,SE=0.78, p<0.01), getting credit, bank loans, or a mortgage (B=-6.86,SE=2.83, p<0.05) and interacting with police or in the courts (B=-4.15,SE=1.48, p<0.01) were associated with lower levels of self-mastery. The findings suggested that discrimination experiences might be harmful by diminishing one’s protective coping mechanism, which warrants longitudinal studies among minority aging populations to clarify.

